# Comparing Perioperative Complications of Off-Clamp versus On-Clamp Partial Nephrectomy for Renal Cancer Using a Novel Energy Balancing Weights Method

**DOI:** 10.3390/life14040442

**Published:** 2024-03-27

**Authors:** Danilo Lofaro, Daniele Amparore, Anna Perri, Vittoria Rago, Alberto Piana, Vincenzo Zaccone, Michele Morelli, Claudio Bisegna, Paolo Pietro Suraci, Domenico Conforti, Francesco Porpiglia, Michele Di Dio

**Affiliations:** 1 Department of Mathematics and Computer Science, University of Calabria, 87036 Rende, Italy; danilo.lofaro@unical.it; 2 Division of Urology, Department of Oncology, School of Medicine, San Luigi Hospital, University of Turin, 10043 Orbassano, Italy; daniele.amparore@unito.it (D.A.); alberto.piana@unito.it (A.P.); francesco.porpiglia@unito.it (F.P.); 3 Department of Experimental and Clinical Medicine, Magna Graecia University, 88100 Catanzaro, Italy; anna.perri@unicz.it; 4 Department of Pharmacy, Health and Nutritional Sciences, University of Calabria, 87036 Rende, Italy; 5 Division of Urology, Department of Surgery, Annunziata Hospital, 87100 Cosenza, Italy; v.zaccone@aocs.it (V.Z.); m.didio@aocs.it (M.D.D.); 6 Department of Obstetrics and Gynecology, Annunziata Hospital, 87100 Cosenza, Italy; m.morelli@aocs.it; 7 Unit of Urological Minimally Invasive Robotic Surgery and Renal Transplantation, Department of Experimental and Clinical Medicine, Careggi Hospital, University of Florence, 50134 Florence, Italy; claudio.bisegna@unifi.it; 8 Urology Unit, Department of Medical-Surgical Sciences and Biotechnologies, Faculty of Pharmacy and Medicine, Sapienza University of Rome, 04100 Latina, Italy; paolopietro.suraci@uniroma1.it; 9 de-Health Lab, Department of Mechanical, Energetic and Management Engineering, University of Calabria, 87036 Rende, Italy; domenico.conforti@unical.it

**Keywords:** partial nephrectomy, hilar clamping, surgical complications, anemia, energy balancing weight, propensity score, minimally invasive surgery

## Abstract

Partial nephrectomy (PN) is the primary surgical method for renal tumor treatment, typically involving clamping the renal artery during tumor removal, leading to warm ischemia and potential renal function impairment. Off-clamp approaches have been explored to mitigate organ damage, yet few results have emerged about the possible effects on hemoglobin loss. Most evidence comes from retrospective studies using propensity score matching, known to be sensitive to PS model misspecification. The energy balancing weights (EBW) method offers an alternative method to address bias by focusing on balancing all the characteristics of covariate distribution. We aimed to compare on- vs. off-clamp techniques in PN using EB-weighted retrospective patient data. Out of 333 consecutive PNs (275/58 on/off-clamp ratio), the EBW method achieved balanced variables, notably tumor anatomy and staging. No significant differences were observed in the operative endpoints between on- and off-clamp techniques, although off-clamp PNs showed slight reductions in hemoglobin loss and renal function decline, albeit with slightly higher perioperative blood loss. Our findings support previous evidence, indicating comparable surgical outcomes between standard and off-clamp procedures, with the EBW method proving effective in balancing baseline variables in observational studies comparing interventions.

## 1. Introduction

Renal cancer represents more than 3% of all newly diagnosed tumors worldwide, with an incidence of more than 400,000 cases per year [1]. Obesity, hypertension, and chronic renal disease are just some of the increasingly prevalent risk factors contributing to the global rise in renal cancer incidence [2]. Consequently, recent diagnostic technological advancements have facilitated the earlier detection of renal lesions in many patients [3]. As a result, 75% of newly diagnosed renal masses are asymptomatic, incidental findings, and typically smaller than 7 cm in diameter [4].

Over the last few decades, there has been growing recognition of the long-term consequences of renal cancer surgery in terms of renal function decline, with constant progress in the knowledge of the etiology of the phenomenon, followed by the reporting of various strategies aimed at minimizing the occurrence of postoperative kidney functional impairment [5,6].

With advancements in minimally invasive surgical techniques, partial nephrectomy (PN) has garnered increased popularity and attention to the point of becoming the standard surgical approach for the treatment of renal tumors nowadays [7]. Several studies have shown that in addition to the benefits of preserving long-term renal function [7,8,9,10], PN may reduce the incidence of cardiovascular events and mortality [8,11,12].

Regardless of the minimally invasive surgical approach (laparoscopic or robot-assisted), the traditional technique of inducing renal warm ischemia by clamping the hilar vessels just before tumor excision remains standard practice. Hilar clamping creates an ischemic state within the kidney, reducing intraoperative blood loss and improving the visibility of the surgical area during tumor removal and subsequent renorrhaphy. Numerous studies indicate that inducing warm ischemia can lead to both short-term and long-term kidney function impairment [13,14]. Consequently, surgeons are advised to minimize warm ischemia time (WIT) whenever possible [15], and strategies of selective clamping have been proposed [16,17].

To further reduce organ damage related to tumor resection, completely off-clamp approaches [18,19] have been developed in recent years.

To date, studies comparing the two techniques have shown no clear advantage in terms of short-term renal function for the off- versus on-clamp approach [20,21,22]. Less evidence has emerged regarding the difference in the incidence of surgical complications, particularly in terms of blood loss and anemia [23,24].

Furthermore, most results come from retrospective observational studies that used propensity score matching (PSM) to reduce possible confounding [25,26,27,28,29,30,31,32,33]. Essentially, the propensity score (e.g., the probability, given the specific patient’s characteristics, of receiving the treatment or the control) acts as a balancing metric: when conditioned on the propensity score, the distribution of observed baseline covariates will be similar (balanced) between the two treatment arms [34]. Beyond PSM, there are other propensity score methods such as inverse probability of treatment weighting (IPTW). IPTW uses the propensity score to estimate a “weight” for each patient, to create a synthetic sample where patient contribution to the outcome estimation is weighted based on the probability of receiving the treatment and the distribution of baseline covariates is independent of this probability [35,36]. Applying either of these methods requires the definition of a model for estimating the propensity score to determine the association between baseline characteristics and received treatment. However, it is known that the misspecification of this model can result in substantial bias in estimating the treatment effect [37]. To address this issue, alternative methods to estimate weights have been proposed that, instead of modeling for the propensity score, are focused on finding a set of weights to maximize baseline variable balancing [38]. In particular, recently, the energy balancing weight (EBW) method was proposed, which estimates weights by minimizing the energy distance (a measure of the difference between two multivariate distributions) between distributions of variables in different treatment groups [39]. The main advantage of the EBW method is that all features of the covariate distribution are balanced, not just means, as with other methods like entropy balancing [40].

Therefore, the aim of our study was to compare the on- and. off-clamp techniques in terms of pre- and post-operative serum hemoglobin changes and intraoperative blood loss in a retrospective cohort of patients undergoing PN statistically weighted using the energy balancing methodology.

## 2. Materials and Methods

### 2.1. Study Population

Data from all patients diagnosed with primary renal cancer who underwent minimally invasive PN from October 2016 to December 2022 at San Luigi Gonzaga Hospital, University of Turin, Italy, were analyzed. Information on age, gender, BMI, Charlson Comorbidity Index (CCI) adjusted by age, comorbidity status, and tumor’s lesion clinical characteristics was collected. Preoperative and first postoperative day serum Hb, creatinine, and eGFR calculated by the Chronic Kidney Disease Epidemiology Collaboration (CKD-EPI) Creatinine equation [41] were recorded. Peri- and postoperative complications, estimated operative blood loss, intraoperative blood transfusion, WIT, surgical time, length of stay, and tumor histotype were collected and analyzed as well. Tumors’ anatomical characteristics were recorded, and the Preoperative Aspects and Dimensions Used for an Anatomical (PADUA) score was calculated [42]. Informed consent was obtained from all the participants. All the investigations were performed according to the principles of the Declaration of Helsinki.

### 2.2. Surgical Technique

All surgical procedures were performed via either robotic or laparoscopic technique by one experienced surgeon, with the choice of transperitoneal or retroperitoneal approaches contingent upon tumor localization, patient demographics, and surgeon discretion. 

During the operation, management of the pedicle showed variability based on the specific characteristics of the renal mass and kidney vasculature, encompassing strategies ranging from off-clamp or selective clamping to the adoption of a global clamping approach.

Tumor excision adhered strictly to the principles of enucleation/enucleoresection. Closure of the renal defect was accomplished using one or two running monofilament sutures, employing diverse techniques dictated by individual surgeon practices. In instances where there was a breach of the urinary collecting system, a specialized suture technique was implemented.

### 2.3. Statistical Analysis

Continuous variables are reported as the mean ± SD or median (IQR) if non-normally distributed, while categorical variables are shown as the frequency (%). To compare baseline data between patients subjected to on- and off-clamp PN, Student’s *t*-test, the Wilcoxon–Mann–Whitney test, the chi-square test, or the Fisher exact test were used as appropriate.

To estimate the EBW, we used the R package WeightIt (v1.0.0) [43]. The model to calculate the weights included the age, gender, BMI, age-adjusted CCI, tumor side, face, clinical size, PADUA score, cystic features, vascular anatomy, clinical T, preoperative Hb, eGFR, and minimally invasive approach. The balance was evaluated using standardized mean differences (SMD), Kolmogorov–Smirnov (KS) statistics, and the variance ratio (for numeric variables). Variables with SMD between −0.1 and 0.1 were considered balanced. The estimand of interest was the average treatment effect in the treated (ATT), which can be considered as the effect of avoiding the treatment of interest (the off-clamp technique in this case) on those who would otherwise receive it [44]. To estimate the marginal effect of the off- vs. on-clamp strategy, the g-computation method was used [45], and confidence intervals were calculated with a bootstrap procedure (999 bootstrap samples). The endpoints considered were the post—preoperative percentage delta for serum Hb and eGFR, the estimated operative blood loss, the length of hospital stay, the total operative time, and the proportion of positive surgical margins. For continuous outcomes, linear regression models were developed to estimate the difference between the surgical approaches, while logistic models were used to estimate the relative risk in the case of categorical variables. Patients undergoing blood transfusion during the surgical intervention were excluded from the analysis of the Hb delta endpoint.

A *p* < 0.05 was considered significant. All analyses were conducted using R version 4.3.2 [46].

## 3. Results

From October 2016 to December 2022, 333 consecutive partial nephrectomies were performed and prospectively registered. Patients’ characteristics at surgery are presented in Table 1. The average patient age was about 64 years, with the majority of patients being males (69%). As expected, on-clamp tumor resections presented a higher PADUA score, with significant differences in lesion size, exophytic rate, and rim location, as well as renal sinus and urinary collecting system (UCS) involvement. Overall, 81% of off-clamp surgeries presented a T1a clinical stage vs. 54% in the on-clamp group (*p* < 0.001), while no significant differences were observed in preoperative Hb and eGFR. The majority of on-clamp PNs were robot-assisted (79% vs. 21% laparoscopic), while this proportion was almost even in the off-clamp group (48 vs. 52%; *p* < 0.001). A similar distribution was observed also for trans versus retroperitoneal access.

In Table 2, the surgical and postoperative characteristics are presented. Differences were recorded both for the postoperative Hb and eGFR loss. The proportion of UCS openings was significantly higher in the on-clamp group, as well as the number of hospitalization days (6.9 vs. 5.8 days for the on- and off-clamp techniques, respectively; *p* = 0.02). In only ten PNs were positive surgical margins recorded, with just one in the off-clamp group. Intraoperative transfusions and other complications were extremely rare, and 94% of the patients were discharged with a Clavien Grade of I or II, with no differences between the different surgical techniques.

**Figure 1:**
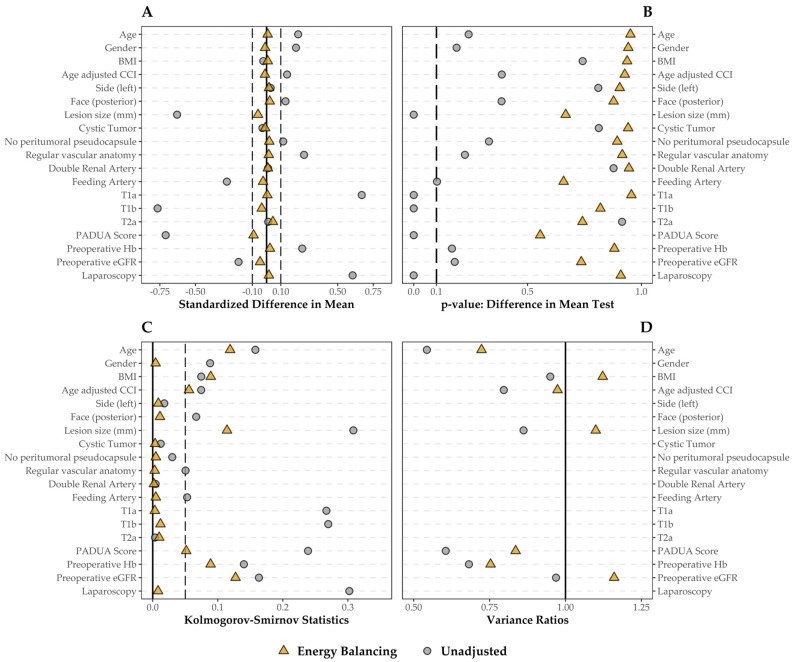
Covariate balance before and after the application of the EBW method; all the categorical covariates included were transformed, creating a binary variable for each category. (**A**) Standardized mean difference: values closer to 0 (solid line) have better balance, values between −0.1 and 0.1 (dashed line) are considered balanced. (**B**) *p*-value for a covariate-by-covariate *t*-test for the differences in means. (**C**) Kolmogorov–Smirnov statistics: the closer to 0 (solid line), the better the balancing, and the 0.05 threshold is indicated as well (dashed line). (**D**) Variance ratios only for numeric variables: optimal value is 1 (solid line), but a value below 2 is considered well balanced.

**Figure 2:**
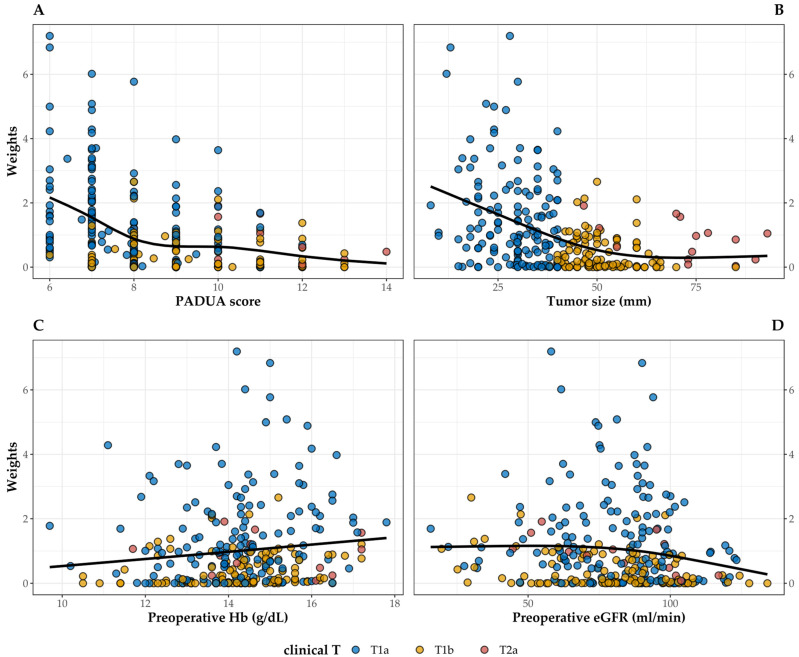
Association between weights assigned by the EBW procedure and PADUA score (**A**), tumor size (**B**), preoperative Hb (**C**), and preoperative eGFR (**D**) in patients in the on-clamp group classified by tumor T staging. The black line shows a smooth cubic spline curve.

To reduce the selection bias and balance the covariates between the two groups, the EBW was calculated, and the derived balancing was checked via SMD. Figure 1 shows the results of the balancing step. All of the variables were balanced, particularly those concerning the tumor’s anatomy and clinical staging presenting a larger SMD before weighting. As it was the minority subgroup, the EBW procedure assigned a weight equal to 1 to all patients in the off-clamp group. To check for possible extreme weights that may have inflated the variance and confidence intervals of the effect estimate, we graphically checked the distribution of the weights assigned to patients in the on-clamp group (median = 0.602, IQR = 0.065–1.379, range = 0–7.195) against the main variables influencing the choice of surgical procedure (Figure 2). As expected, there was a negative significant association between weights and PADUA score (R^2^ = 0.138, *p* < 0.001), as well as tumor size (R^2^ = 0.164, *p* < 0.001). The associations with preoperative Hb (R^2^ = 0.011, *p* = 0.041) and preoperative eGFR (R^2^ = 0.009, *p* = 0.059) were weaker.

None of the analyzed endpoints showed statistically significant differences between the different surgical procedures (Table 3). The estimated average effect of the off-clamp technique was a 2.3% reduced Hb loss (95% CI −1.4–6.2) and a 6.7% reduced eGFR loss (−3.9–17.4). Similarly, off-clamp PNs resulted in a higher average operative blood loss of 25.1 mL (−39.7–88.0), −0.54 hospitalization days (−1.5–0.5), and essentially no difference in the average risk of positive surgical margins (RR 1.16, 95% CI 0.16–5.50).

## 4. Discussion

Taken together, our results highlight how there are no significant differences between on- and off-clamp PN in terms of perioperative complications and confirm the EBW method as a reliant statistical technique for covariate balance in the context of causal effect estimation in observational studies.

In our study, no differences in Hb loss were observed between the two surgical procedures. Even though hemorrhagic complications have been an important focus in the literature on the matter, few studies have presented data on comparing postoperative Hb decline. We found a non-significant difference of around 2% Hb delta in favor of off-clamp PNs, similar to that presented in previous studies. In a two-center retrospective study, Bertolo et al. [31] analyzed 600 patients from two distinct high-volume centers and found a 0.2 g/dL difference in Hb loss after PS matching, though the authors did not estimate the endpoint via statistical modeling. More similarly to our finding, the CLOCK II trial [22] found no difference in post-operative Hb between on- and off-clamp laparoscopic PN, although interestingly, this difference was larger on the fifth postoperative day than on the first. 

This result is confirmed by the absence of any significant difference in estimated blood loss in our cohort. Previous observational studies found significantly higher blood loss in off-clamp PNs [26,30], although the average difference in both cases was around 50 mL. On the other hand, all the RCTs presented to date confirm our results [20,21,22].

Even though the main objects of our work were surgical complications, we evaluated the difference in postoperative renal function as well. As expected, the eGFR loss was similar in the two subgroups, as emerged in the most recent literature. The systematic review by Shrivastava and colleagues [47], pulling results from two RCTs and nine PS-matched observational studies and a total of 2483 patients undergoing robotic PN, found no difference whatsoever in early renal function decline between the two surgical procedures, and similar findings were reported by Bertolo et al. [22] in laparoscopic PNs. Recent evidence highlights how the association between ischemia and postoperative eGFR is influenced by baseline renal function [48], and only values of WIT longer than thirty minutes seem to be predictive of a transition to acute kidney disease [49]. On the other hand, there is a growing body of literature about the role of residual parenchymal volume as the predominant factor in the conservation of kidney functionality [50,51], and the recent work by Xiong and colleagues, which found no association between ischemia time and histological modification in healthy renal parenchyma, seems to confirm these observations. 

The only discrepancies between our results and those of the meta-analysis by Shrivastava concern the total operative time and the incidence of positive margins [47]. In their systematic review, these authors found a significant difference of almost twenty-two minutes in favor of off-clamp PNs, but it should be noted that this result seems to be highly influenced by a study limited to cT2 tumors which reported a massive difference of 80 vs. 190 operative minutes in favor of the off-clamp technique [32]. Regarding the incidence of positive margins, our results are in line with previous PS-matched studies where no significant effects were registered, while the pulled results evidenced a slight, but significant, reduction in the incidence in the off-clamp group. In this case, additional data are necessary to assess whether the meta-analysis results were affected by the heterogeneity of the selected studies or whether there is an actual beneficial effect in not clamping the renal pedicle. Nevertheless, both the latter outcomes discussed are highly dependent on the experience of the operative surgeons, and comparisons between different cohorts might be inaccurate.

It is worth noting that the classification system for assessing the surgical complexity of renal masses relies on outdated nephrometric scores that fail to incorporate the latest advancements in diagnostic imaging technology. For example, the emergence of new three-dimensional reconstructions derived from 2D images enables the precise characterization of the kidney’s vasculature, facilitating the planning of selective clamping or clampless procedures with enhanced safety measures. As a result, certain renal masses may be deemed less surgically complex than indicated by traditional classification methods. Looking ahead, there is a need to overhaul nephrometric scores to better capture the true surgical complexity of renal neoplasms [52,53,54].

In our study, we used a novel statistical technique to balance the distribution of covariates across treatments and control for potential bias. In their work, Huling and Mak demonstrated how the main source of bias for the estimation of average effects in the context of observational study is covariate imbalance [39]. Unlike other methods, the advantage of their EBW method lies in balancing all characteristics of the variable distribution, not only mean and variance. In line with their findings, EBW application in our cohort resulted in a good covariate balance between on- and off-clamp groups, in particular for those variables known to be highly influential for the choice of the surgical approach, such as the PADUA score, clinical tumor staging, and baseline eGFR. In particular, we ran balance checking diagnostics using SMD, KS statistics, and the variance ratio for continuous variables as per best practice in studies based on the propensity score [55]. Comparison with previous analyses is difficult, since ours is the first using weighting instead of PS matching [25,26,27,28,29,30,31,32,33], and in these studies, balance checking, when reported, is often based on hypothesis testing (e.g., t-test, Mann–Whitney test, or chi-square test), which has been demonstrated to be potentially misleading [56,57].

We chose ATT as the estimand to investigate. Since the focus of our work was the evaluation of differences in surgical complications between the two techniques, our primary research question was whether patients undergoing off-clamp partial nephrectomy would benefit from the “classical” procedure in terms of perioperative safety. The use of g-computation for the ATT estimate [58] allowed for the evaluation of the “marginal” effect of off-clamp PN, namely the average effect of treatment on the whole population and not on a single patient, while with the bootstrap procedure, we were able to obtain robust standard errors and confidence intervals [59]. Given the reliability of the statistical methods applied, we think that the reported results could be generalized to the population from which our cohort was derived, which is represented by patients who are candidates for minimally invasive PN, with relatively small, non-complex tumors and preserved renal function.

This study has limitations worth considering. First, the observational nature of the study does not allow us to exclude the existence of additional confounding not accounted for, even if the statistical methodology used assures the minimization of potential bias. In addition, this study lacks the evaluation of markers more indicative of kidney damage than the eGFR. As evidenced in a recent study [60], serum and urinary biomarkers could help in the identification of patients at a higher risk of long-term renal dysfunction. In addition, our patients were enrolled over a relatively long time range (six years); therefore, the surgeons’ increasing level of experience may have influenced the incidence of operative complications.

## 5. Conclusions

In conclusion, through the implementation of a different statistical method than the majority of the published contributions, we confirmed how the off-clamp approach for partial nephrectomy has no effect in terms of the incidence of major intraoperative complications when compared to the “traditional” technique, and at the same time no advantages in terms of the early recovery of kidney functionality. Future studies should focus on identifying factors affecting renal function and chronic renal disease onset in the long term to define the best overall treatment approach for each specific patient.

## Figures and Tables

**Table 1:** Patients’ characteristics at baseline.

Characteristic	Overall *n* = 333	On-Clamp *n* = 275	Off-Clamp *n* = 58	*p*-Value
Age (years)	63.87 ± 12.45	63.49 ± 12.98	65.66 ± 9.50	0.144
Gender (Male)	229 (69%)	185 (67%)	44 (76%)	0.2
BMI (Kg/m^2^)	25.80 ± 3.96	25.81 ± 3.98	25.71 ± 3.91	0.857
CCI (age-adjusted)	4 (3–5)	4 (3–5)	4 (3–5)	0.465
Solitary Kidney	15 (4.5%)	11 (4.0%)	4 (6.9%)	0.307
Side				0.99
Right	172 (52%)	142 (52%)	30 (52%)	
Left	161 (48%)	133 (48%)	28 (48%)	
Face				0.125
Anterior	202 (61%)	172 (63%)	30 (52%)	
Posterior	131 (39%)	103 (37%)	28 (48%)	
Polar location				0.508
Inferior	90 (27%)	77 (28%)	13 (22%)	
Middle	150 (45%)	120 (44%)	30 (52%)	
Superior	93 (28%)	78 (28%)	15 (26%)	
Exophytic rate				0.004
≥50%	165 (50%)	125 (45%)	40 (69%)	
<50%	125 (38%)	110 (40%)	15 (26%)	
Endophytic	43 (13%)	40 (15%)	3 (5.2%)	
Rim location				0.006
Lateral	212 (64%)	166 (60%)	46 (79%)	
Medial	121 (36%)	109 (40%)	12 (21%)	
Renal sinus involvement	70 (21%)	67 (24%)	3 (5.2%)	0.001
UCS involvement	66 (20%)	62 (23%)	4 (6.9%)	0.007
Tumor size score (cm)				0.001
≤4	194 (58%)	148 (54%)	46 (79%)	
4.1–7	127 (38%)	116 (42%)	11 (19%)	
>7	12 (3.6%)	11 (4.0%)	1 (1.7%)	
Padua Score	8 (7–9)	8 (7–10)	7 (7–8)	<0.001
Tumor Size (mm)	39.13 ± 15.67	40.79 ± 15.50	31.24 ± 14.11	<0.001
Cystic tumor	49 (15%)	41 (15%)	8 (14%)	0.827
Peritumoral pseudocapsule	318 (95%)	264 (96%)	54 (93%)	0.307
Vascular anatomy				0.160
Regular	307 (92%)	251 (91%)	56 (97%)	
Double renal artery	11 (3.3%)	9 (3.3%)	2 (3.4%)	
Feeding artery	15 (4.5%)	15 (5.5%)	0 (0%)	
Clinical T				<0.001
T1a	195 (59%)	148 (54%)	47 (81%)	
T1b	121 (36%)	113 (41%)	8 (14%)	
T2a	17 (5.1%)	14 (5.1%)	3 (5.2%)	
Preoperative Hb (g/dL)	14.29 ± 1.44	14.23 ± 1.48	14.56 ± 1.20	0.078
Preoperative eGFR (mL/min)	79.46 ± 21.71	80.21 ± 21.73	75.88 ± 21.41	0.166
Minimally invasive approach				<0.001
Robot-assisted	246 (74%)	218 (79%)	28 (48%)	
Laparoscopic	87 (26%)	57 (21%)	30 (52%)	
Access				<0.001
Transperitoneal	249 (75%)	219 (80%)	30 (52%)	
Retroperitoneal	84 (25%)	56 (20%)	28 (48%)	

CCI: Charlson Comorbidity Index; UCS: urinary collecting system.

**Table 2:** Patients’ surgical and postoperative characteristics.

Characteristic	Overall *n* = 333	On-Clamp *n* = 275	Off-Clamp *n* = 58	*p*-Value
Postoperative Hb (g/dL)	12.18 ± 1.50	12.07 ± 1.51	12.70 ± 1.39	0.002
Post—Preoperative Hb Delta (%)	−14.46 ± 9.48	−14.86 ± 9.70	−12.57 ± 8.18	0.065
Postoperative eGFR (mL/min)	70.94 ± 23.90	71.07 ± 24.21	70.36 ± 22.60	0.831
Post—Preoperative eGFR Delta (%)	−10.84 ± 17.47	−11.67 ± 17.84	−6.91 ± 15.15	0.038
Warm Ischemia Time (min)	17.44 ± 7.59	18.58 ± 7.38	0 ± 0	<0.001
Imaging support	125 (38%)	115 (42%)	10 (17%)	<0.001
UCS Opening	62 (19%)	61 (22%)	1 (1.7%)	<0.001
Estimated blood loss (mL)	187.39 ± 187.44	189.45 ± 170.39	177.61 ± 254.86	0.736
Intraoperative transfusion	4 (1.2%)	3 (1.1%)	1 (1.7%)	0.537
Post-operative complications	4 (1.2%)	4 (1.5%)	0 (0%)	>0.999
Clavien Grade				0.569
I	286 (86%)	234 (85%)	52 (90%)	
II	26 (7.8%)	21 (7.6%)	5 (8.6%)	
III	19 (5.7%)	18 (6.5%)	1 (1.7%)	
IV	1 (0.3%)	1 (0.4%)	0 (0%)	
V	1 (0.3%)	1 (0.4%)	0 (0%)	
Length of stay (days)	6.70 ± 3.08	6.88 ± 3.23	5.84 ± 2.00	0.002
Malignant	272 (82%)	228 (83%)	44 (76%)	0.207
ISUP Grade				0.622
I	41 (12%)	35 (13%)	6 (10%)	
II	187 (56%)	156 (57%)	31 (53%)	
III	41 (12%)	35 (13%)	6 (10%)	
IV	1 (0.3%)	1 (0.4%)	0 (0%)	
N.A.	63 (19%)	48 (17%)	15 (26%)	
Necrosis	68 (20%)	57 (21%)	11 (19%)	0.762
Positive Surgical Margins	10 (3.0%)	9 (3.3%)	1 (1.7%)	>0.999
pT				<0.001
benign	35 (11%)	24 (8.7%)	11 (19%)	
pT1a	159 (48%)	124 (45%)	35 (60%)	
pT1b	91 (27%)	84 (31%)	7 (12%)	
pT2	9 (2.7%)	8 (2.9%)	1 (1.7%)	
pT3a	38 (11%)	35 (13%)	3 (5.2%)	
pT3b	1 (0.3%)	0 (0%)	1 (1.7%)	

UCS: urinary collecting system.

**Table 3:** ATTs of the outcomes analyzed.

	ATT	95% CI	*p*-Value
Post—Preoperative Hb Delta (%) *^‡^	2.347	−1.375–6.195	0.210
Post—Preoperative eGFR Delta (%) *	6.731	−3.861–17.369	0.213
Perioperative estimated blood loss (mL) *	25.124	−39.73–88.023	0.433
Total Operative time (min)	−8.369	−19.77–3.176	0.152
Length of stay (days) *	−0.541	−1.501–0.484	0.270
Positive Surgical Margins ^†^	1.157	0.163–5.507	0.873

* ATT expressed as mean difference between the off- and on-clamp techniques; ^†^ ATT expressed as relative risk of off- with respect to on-clamp techniques; ^‡^ analysis of 329 patients with no intraoperative transfusion.

## Data Availability

The data can be shared up on reasonable request.
